# Non-Adaptive Phenotypic Evolution of the Endangered Carnivore *Lycaon pictus*

**DOI:** 10.1371/journal.pone.0073856

**Published:** 2013-09-23

**Authors:** Charles T. T. Edwards, Gregory S. A. Rasmussen, Philip Riordan, Franck Courchamp, David W. Macdonald

**Affiliations:** 1 Division of Biology, Imperial College London, Silwood Park, Ascot, SL5 7PY, United Kingdom; 2 Wildlife Conservation Research Unit, Department of Zoology, University of Oxford, Tubney House, Tubney, Oxon, United Kingdom; 3 Painted Dog Research Project, Natural History Museum, Bulawayo, Zimbabwe; 4 Ecologie, Systématique & Evolution, UMR CNRS 8079, Université Paris-Sud, Orsay, France; Bangor University, United Kingdom

## Abstract

Decline in wild populations as a result of anthropogenic impact is widely considered to have evolutionary consequences for the species concerned. Here we examine changes in developmental stability in the painted hunting dog (*Lycaon pictus*), which once occupied most of sub-Saharan Africa but has undergone a dramatic population decline in the last century. Fluctuating asymmetry (FA) was used as an indicator of developmental stability and measured in museum skull specimens spanning a hundred year period. A comparison with the more ubiquitous black-backed jackal (*Canis mesomelas*) revealed FA in *L. pictus* to be high. Furthermore, the data indicate a temporal increase in FA over time in *L. pictus*, corresponding to the period of its population decline. The high rate of change is compatible with genetic drift although environmental factors are also likely to be important. Lowering developmental stability over time may have direct fitness consequences and as such represents an unacknowledged threat to future resilience of the population.

## Introduction

Anthropogenic impacts on biological processes are believed to be a major driver of evolution in affected populations over both long-term and contemporary, measurable time scales. The latter can occur within a few hundred generations [1], [2] as populations seek to adapt to rapid changes in their environment [3], . Adaptive evolution usually refers to that which takes place in response to natural selection – differential survival in the prevailing environment and associated heritable genetic variation – and makes an important contribution to population persistence when faced with environmental change [5]–[7]. However, adaptation may also be phenotypic, with the phenotype exhibiting a degree of plasticity in response to prevailing environmental conditions.

Plasticity is itself a heritable trait subject to natural selection and can confer the genotype with a degree of resilience through better matching of the phenotype and the environment [8], [9]. This is particularly useful when conditions fluctuate within a prescribed range. However plasticity must be balanced by the need to maintain functional integrity of the organism as the environment moves beyond conditions historically experienced by the population (environmental *stress*). Environmental change of this magnitude will challenge ability of the individual to maintain homeostasis and the organism must buffer itself against environmental stress so that proper development and function can still occur [9]–[11].

Under extreme conditions, non-adaptive plasticity in response to stress may reect a fundamental breakdown during phenotypic development or disruption of physiological function [12]. As an organism develops it is subject to micro-environmental perturbations and the degree to which these perturbations affect the developmental process and increase phenotypic variation is referred to as developmental stability (DS), or environmental canalisation [13]. Compared to adaptive plasticity, a lowered DS arises as a passive consequence of environmental stress and is better viewed as a failure of compensatory mechanisms [14].

Non-genetic variation in every trait is influenced by DS, but it is most intuitively inferred from the symmetrical development of bilateral traits. Non-directional departures of paired structures from perfect symmetry, termed fluctuating asymmetry (FA), can be used to measure DS [15]–[19], with a high degree of FA representing an obvious departure from the optimal developmental trajectory of an organism. Fluctuating asymmetry arguably demonstrates that developmental homeostasis has been compromised and has been frequently reported in wild populations experiencing environmental stress (e.g. [20]–[25]), although inconsistent results have prevented its widespread use in conservation [26], [27]. The underlying physiological mechanisms behind DS remain unclear but empirical studies continue to report evidence of its heritability under certain conditions (e.g. [28], [29]), suggesting some genetic influence [15].

Empirical studies that have identified FA in wild populations have predominantly used contemporary controls for inference [20]–[24], [30]. In this study we were able to measure changes in FA over time based on historic museum specimens of the endangered painted hunting dog (*Lycaon pictus*). This represented a unique opportunity to correlate FA with environmental change and population decline, emphasising the importance of museum collections to population biology.

As a monotypic genus, *Lycaon* ranks as a high conservation priority, and a dramatic population decline, precipitated by culling [31]–[33], has made it endangered [34]. *L. pictus* populations formerly occupied most of sub-Saharan Africa, but of the thirty-four countries in its former range it has been extirpated from nineteen and is now confined to comparatively small populations in southern and east Africa [34]. Following a 99% reduction [35] numbers are estimated to be around 6,600 individuals [34], with many populations both inside and outside protected reserves still in decline as a result of persecution and degradation of their former habitat [34], [36], [37]. Samples collected over a 100-year period allowed us to measure temporal changes in FA and thus examine the potential for anthropogenically driven non-adaptive evolution, with clear implications for carnivores facing similar threats across Africa.

## Materials and Methods

### Data

*Lycaon* skulls were accessed from the Natural History Museum Zimbabwe (103), Ditsong National Museum of Natural History (25) and Kruger National Park (5). These sources held a total of 133 skulls collected between 1913 and 2001, originating from Zimbabwe (38), Botswana (14), Zambia (53) and South Africa (28), and representing a continuous region of south east Africa. Permission to conduct research in Zimbabwe was obtained from the Director General of the Parks and Wildlife Management Authority, with access to the collection of the Natural History Museum of Zimbabwe permitted by Albert Kumerai. Permission was also obtained from the Ditsong National Museum of Natural History, Pretoria and South African National Parks for access to their collections. All specimens were accessed temporarily for the purposes of this study with measurements and photographs taken on site.

Twenty paired calliper measurements were made (Table S1) following the methodology of Wayne *et al*. [38] and compared to measurements taken from the black-backed jackal (*Canis mesomelas*), which acted as a sympatric control group. A total of 102 *Canis* specimens collected between 1949 and 2000 were measured, originating from Botswana (66) and Zimbabwe (36). To better establish the existence of a temporal trend in FA, *Lycaon* skulls were further measured using photogrammetric methods (Figure S1). This independent duplication, shared between the primary authors, was designed as a novel guard against measurement error or observer bias in temporal analyses of FA. All samples were selected and measured blind with respect to the year of collection, to prevent systematic changes in measurement error potentially yielding a spurious result.

### Statistics

To estimate DS it is necessary to calculate *ideal* FA from a sample population, defined as a normal distribution with zero mean of the left minus right difference between measurements of a bilaterally symmetrical trait [17]. Other developmental processes that may complicate this calculation include antisymmetry, directional asymmetry and allometry. Both anti- and directional asymmetries occur when two sides of a bilateral character consistently differ in size. In the case of antisymmetry, the largest side varies randomly among individuals [17]. Allometry refers to the size dependence of asymmetry and is an important consideration when estimating FA from a population of individuals of different size. To correct for size dependence, FA for individual *i* and trait *j* was measured as the difference between left (*L_ij_*) and right (*R_ij_*) measurements divided by the average size [39]:
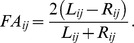



Following Palmer [40] and Palmer and Strobeck [39] we tested for departures from ideal FA using three statistical tests of the distribution of *FA* for each trait, namely a two-tailed *t*-test of the mean against zero and *z*-tests of platykurtosis (indicating a flat or bimodal shape) and skew [41]. Skew in either direction provides evidence of directional or antisymmetry and is ground for exclusion, so this test was also two-tailed. Kurtosis is a measure of antisymmetry, and as we were only interested in detecting negative departures from normal kurtosis (defined as equal to zero), this test was one-tailed. This is because leptokurtosis (kurtosis values greater than zero) could be a consequence of sampling from more than one population with different degrees of FA (i.e. different variances in the *FA* distribution), and as such would not indicate a departure from ideal FA [42], [43]. To correct the overall Type I error for multiple tests, *p*-values were adjusted using the sequential Bonferroni procedure [41], with traits excluded on the basis of adjusted *p*-values at a significance level of 0.1. Traits excluded from further analyses are listed in Tables S1 and S2. In total, four traits were removed from the photogrammetric dataset, and one trait from the calliper dataset.

Subsequent analyses were conducted on the absolute FA values, *|FA|* [39]. We also calculated a composite index for each individual (*CFA*), as the mean *|FA|* across traits. Combining multiple traits in this way may provide a more accurate measure of FA [39], [44], assuming a consistent overall level of DS for a particular individual.

A linear model was used to measure changes in FA over time. To reduce heterodescasity, the *|FA|* values were log-transformed, in common with many studies of evolutionary rate in morphological characteristics [45]–[47]. Sex differences in FA were tested but found not to be significant and the sexes were therefore pooled. Similarly we found no evidence for a difference in FA between samples collected inside and outside of protected areas. Based on unquantified regional differences in demography and environmental conditions, we included a *country* effect, as well as an interaction between *year* and *country*. Because of repeated measures on each individual, *individual* was considered a random effect (*α*). The measured characters were considered an arbitrary subset of morphological traits and so *character* was also a random effect (*β*). Since multiple characters were measured per individual, *character* and *individual* represent an unbalanced crossed factorial design, giving the linear mixed-effects model:
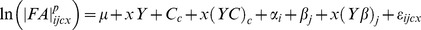
with *year* (*Y*), *country* (*C*) and the interaction *(YC)* as fixed effects, for individual *i = 1,…,m*; character *j = 1,…,n*; country *c*  =  [Botswana, Zimbabwe, Zambia, South Africa]; year *x = 1913,…,2001*; and method *p* (photogrammetric or calliper). For simplicity of presentation, each method was analysed separately, although a combined analysis yielded the same result. The random effects, including the error term (*ε*), were assumed to be normally distributed. The significance of *character*, *year*, and the interaction between *year* and *character* (*Yβ*), were tested using the likelihood ratio statistic, with *p*-values calculated through comparison with a null distribution generated by parametric bootstrap of the null model [48]. We assumed *a priori* that FA would be different in each country and therefore retained the *country* effect (and interaction between *country* and *year* where appropriate) in the final model, irrespective of apparent significance. Although this risks over-fitting the data and could lead to less powerful tests of significance, it allowed us scope to infer both an overall rate (from the *Y* coefficient) and the rate of change in each country (from *Y + (YC)_c_*).

Again assuming country specific differences we next tested for significance of *year* using a fixed effects model for the composite FA index:
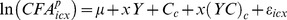
with standard methods described in Faraway [49].

We further sought to quantify the rate of change in evolutionarily relevant units. Following recommendations by Hendry and Kinnison [45], we measured evolutionary rate by performing a linear regression of the Haldane:
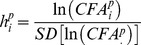
against the number of elapsed generations, where *SD[.]* is the standard deviation across the sample of individuals [47]. We assumed generation lengths specific to each country: Zimbabwe –5.7 years [34], South Africa –6.2 years [50], Botswana –4.5 years [50], Zambia –5.0 years [34]; estimated from empirical observation as the average age of reproductive females (with the exception of Zambia, for which we used an approximate reference value). The Haldane is more relevant as a rate measure since it scales the magnitude of change by the degree of phenotypic variation that exists in a population [45], [51], and furthermore allows comparison with other studies (e.g. [46]).

## Results

**Figure 1:**
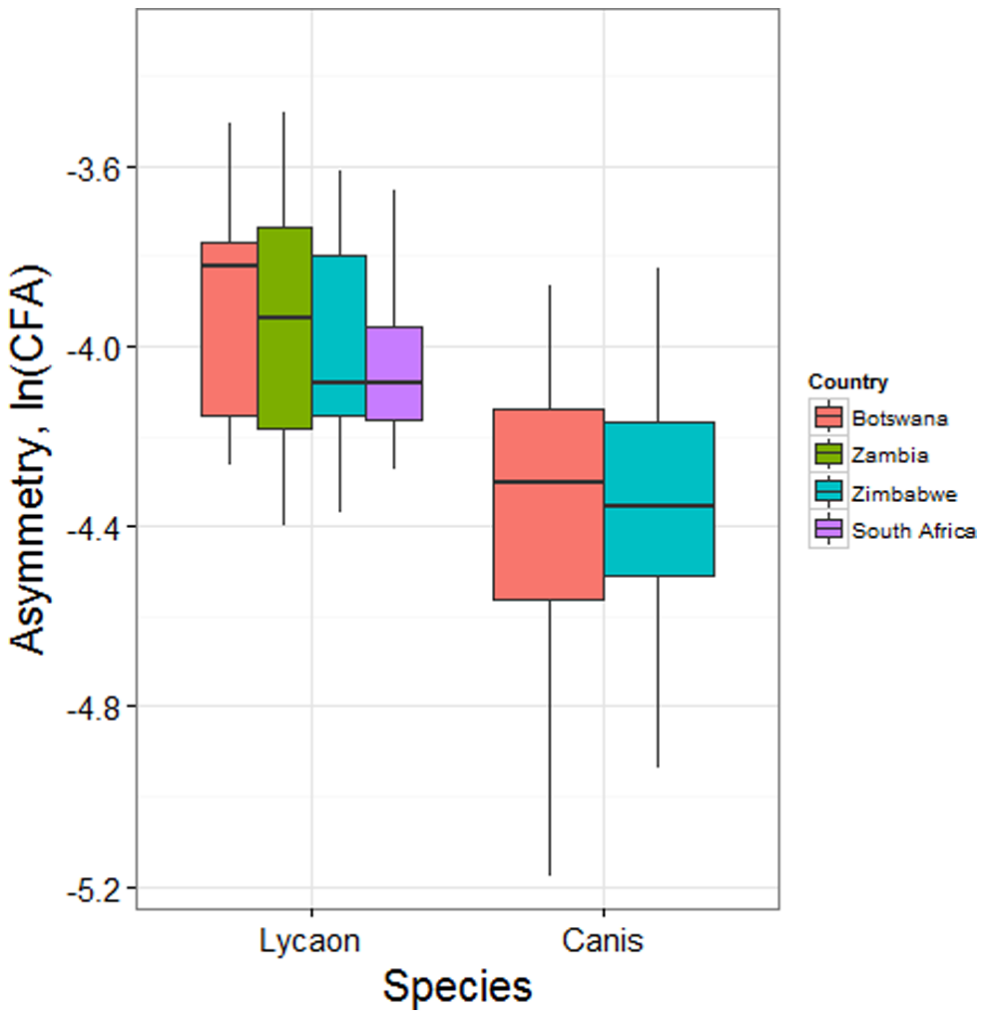
Comparison of asymmetry *ln (CFA)* in *Lycaon pictus* and *Canis mesomelas* between 1949 and 2000. An analysis of variance with a sample size *n* = 186 yielded a significant difference (*p*<0.0001) between species but not between countries (*p* = 0.629).

Following the exclusion of characters that did not exhibit ideal FA (Tables S2 and S3), we first compared *L. pictus* to *C. mesomelas* to examine whether the former exhibited unusually high asymmetry. *C. mesomelas* occupies a similar spatial distribution but has not undergone a population contraction of the magnitude experienced by *L. pictus* [52], making it a good control group. An analysis of variance showed FA in *L. pictus* to be significantly higher when estimated using the composite measure (Figure 1), with a similar pattern for individual traits (Figure S2), giving a first indication that FA may be increased by reductions in population size.

**Figure 2:**
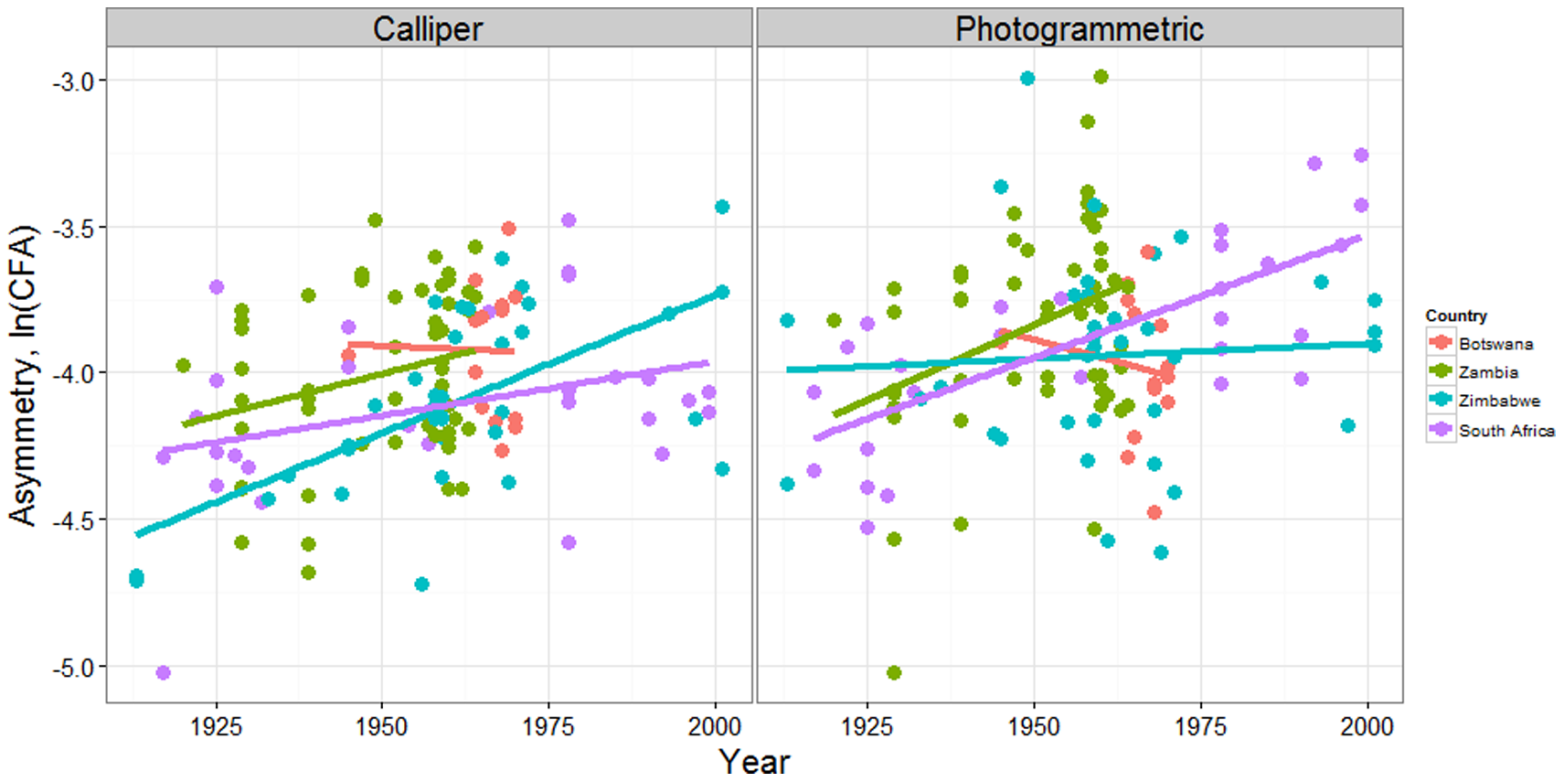
Fluctuating asymmetry *ln (CFA)* over time for calliper and photogrammetric measurements, with linear regression lines shown for each country.

We next measured the rate of phenotypic change over time in cranial asymmetry of *L. pictus* using both manual callipers and photogrammetric measures, again first testing for ideal FA (Tables S3 and S4). Plots of the log-transformed FA data are given in Figure S3. Using a mixed effects linear model of the data collected for each method, the overall rates of change over time were found to be significantly greater than zero (Table 1). Country specific estimates were all positive with the exception of Botswana. The significance of *year* and *country* fixed effects are given in Table S5 with model diagnostics in Figure S4. For both calliper and photogrammetric methods the *character* random effect was highly significant (*p*<0.0001) but the interaction between *character* and *year* was not, and was omitted from the final model. Combining data from multiple characters into a composite measure of FA for each method, a linear regression further confirmed significant evidence for a positive increase over time (Figure 2, Table 2, Figure S5 and Table S6). Measured in Haldane's, for callipers and photogrammetric measures, evolutionary rates of 0.085 and 0.060 standard deviations per generation respectively, were observed (Table 2).

**Table 1:** Estimated rates of change in fluctuating asymmetry from mixed effects models of *ln (2|L* *−* *R|/(L+R))* over time for photogrammetric and calliper measures.

Method	Country	*Ln (|FA|)*
		per year
Callipers	Botswana	−0.0040
	Zambia	0.0050
	Zimbabwe	0.0063
	South Africa	0.0050
	**Overall**	0.0031
	*p-value*	*0.0080*
Photogrammetric	Botswana	−0.0031
	Zambia	0.0082
	Zimbabwe	0.0029
	South Africa	0.0084
	**Overall**	0.0041
	*p-value*	*0.0020*

Rates of change per country were obtained by assuming an interaction between time and country. The overall rate of change refers to the year coefficient *Y*, which can be considered an average across countries. The significance of this term is given as a *p*-value.

**Table 2:** Rate of change per year in cranial asymmetry measured using composite fluctuating asymmetry *ln (CFA)* with respect to time, and Haldanes with respect to generations.

Method	Country	*Ln (CFA)*	Haldanes
		per year	per generation
Callipers	Botswana	−0.0010	−0.015
	Zambia	0.0058	0.097
	Zimbabwe	0.0094	0.181
	South Africa	0.0037	0.077
	**Overall**	0.0045	0.085
	*p-value*	*<0.0001*	*0.0005*
Photogrammetric	Botswana	−0.0060	−0.081
	Zambia	0.0101	0.150
	Zimbabwe	0.0010	0.017
	South Africa	0.0084	0.154
	**Overall**	0.0034	0.060
	*p-value*	*0.0005*	*0.0002*

Rates of change per country were obtained by assuming an interaction between time and country. The overall rate of change refers to the *year* or *generation* coefficient and can be considered an average across countries. Significance of the time coefficient (in years or generations) from each regression is shown as a *p*-value.

**Figure 3:**
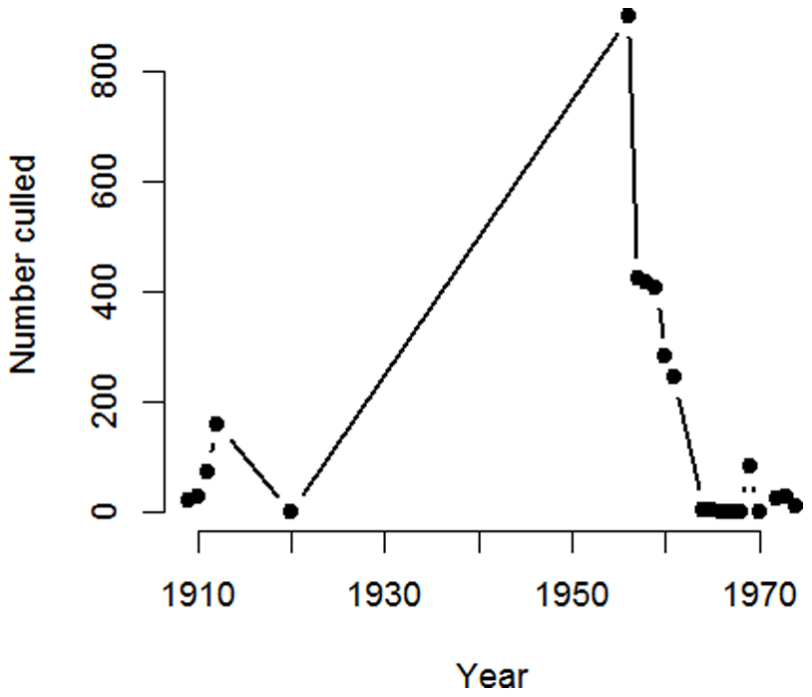
Number of *Lycaon pictus* recorded as killed by hunters in Zimbabwe between 1909 and 1974.

The phenotypic evolution observed took place during a time of rapid population decline and habitat degradation [37], [53]. Although this decline has not been quantified precisely, we know that *Lycaon* was considered vermin and subjected to widespread persecution in southern Africa, including culling programmes [31]–[33], [54]. Records only occur in some areas, but data were available for Zimbabwe [31], [55], which is representative of the region. In Zimbabwe, culling of *L. pictus* was formally intensified in the 1950s, when bounty hunters were supplemented by personnel specifically employed for the job. This intensified effort led to diminishing culling returns post 1960, attributable to dwindling numbers of *L. pictus* despite continued intensive effort by the hunters [31]. A drop in population can therefore be inferred from a drop in the numbers killed, which is illustrated in Figure 3.

## Discussion

The overall increase in FA in *L. pictus* observed during a period of population decline and habitat fragmentation corroborates the high levels of asymmetry in other species suffering similarly, notably birds (e.g. [22]–[24]), but also other carnivores (e.g. [38], [56]). Increased FA has further been observed in island populations of mammals, which are notably low in genetic diversity [57]–[59], and captive populations of endangered ungulates [60]–[62]. Although the causal mechanisms behind these observations are unclear, we discuss the potential role of genetic factors.

Evidence of declining DS illustrates that phenotypic evolutionary change need not always be adaptive. One genetic process that can undermine adaptation is genetic drift, which can alter genotypic frequencies independently of their survival benefit, and lead to a loss of genetic diversity [63]. Genetic drift is pertinent to small populations of high conservation priority and has received a great deal of attention in the conservation literature because of the threat it represents to long-term viability [64], [65]. Although studies of neutral genetic diversity in *L. pictus* suggest it to be comparatively unremarkable [66]–[68], non-neutral loci reveal a high degree of homogeneity [69] and exhibit genetic signatures of demographic contraction and fragmentation of the population [70]. The fragmented structure of remnant populations, their small numbers and low densities makes genetic drift a concern, and to examine this further we used a measure suggested by Lynch [51] to compare an observed evolutionary rate with that expected by drift:
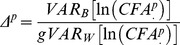
where *g* is the number of generations and *VAR_B_[.]* and *VAR_W_[.]* are the between- and within-generation variance. These were obtained from the linear regression of *ln(CFA^p^)* against year. Lynch [51] proposed a neutral expectation for mammals with higher and lower values consistent with diversifying or stabilising selection respectively. With regards to DS, we would expect stabilising selection to produce Δ values less than Δ_min_  = 1.0e-4. However for *L. pictus*, we estimated Δ = 7.5e-4>Δ_min_ for both photogrammetric and calliper measurements. Although this observation is far from conclusive, it suggests that our observations of evolutionary change are at least compatible with drift and a failure of stabilising selection for DS.

Despite earlier suggestions that demographic threats make genetics an unnecessary consideration [71], [72], subsequent analyses of empirical data have demonstrated that genetic drift and associated low levels of genetic diversity can result in reduced population fitness [73]. Proposed causal mechanisms include the accumulation of deleterious mutations [74], [75], a loss of adaptive potential [76] and inbreeding depression [77], the last being a consequence of homozygous expression of deleterious alleles. Of these, inbreeding depression is most likely to have an impact over time scales relevant to conservation [64], and empirical evidence has demonstrated its occurrence in wild populations [78]–[80]. Although evidence of an increased extinction risk associated with inbreeding is anecdotal [81], a causal link is well supported by both theoretical arguments [64], [82]–[84] and laboratory studies (e.g. [85]).

There is some empirical evidence for increased FA in inbred populations with reduced reproductive fitness [60]–[62]. However, since being first suggested by Lerner [86], who proposed that a degree of genetic redundancy would improve developmental homeostasis in a fluctuating environment, the relationship between homozygosity and DS has proved controversial [15], [87]. Despite early promise that FA may provide a useful tool for conservation genetics [88], empirical studies have largely failed to detect heritable variation [89], and laboratory studies relating homozygosity to FA have produced inconsistent results [27]. This is likely due in part to a weak statistical relationship between FA and DS [87], which undermines the power of studies that use FA to identify causal mechanisms behind DS [27]. Any association between inbreeding and developmental stability is therefore difficult to detect even when the level of inbreeding is high [90].

Although previously cited evidence suggests a link between reduced population size and increased FA, whether this is environmental or genetic in origin remains unclear. We note that the population size of *L. pictus* is well within the range at which genetic drift will start to have an homogenizing influence [91], a conjecture supported by empirical data [70], but despite extensive study there is currently no phenotypic evidence for inbreeding depression in wild populations. Since both environmental and genetic factors can act in concert to reduce DS [15], [16], we hypothesise that a combination of reduced genetic diversity as a result of population contraction and fragmentation, and habitat degradation has disrupted developmental homeostasis progressively over time, driving the increase in asymmetry reported here. Instability of the phenotype may itself have direct fitness consequences and therefore represents an unacknowledged threat for the viability of remnant *L. pictus* populations, as well as other large carnivores in Africa faced with similar conservation concerns.

## Supporting Information

Figure S1 **Photogrammetric measurements taken on** ***Lycaon pictus*** **skulls.** Images were taken using a skull stand and spirit level, and eighteen paired photogrammetric measurements were taken from the underside of the cranium and analysed using Adobe Photoshop image software.
(TIF)

Figure S2 **Comparison of log-transformed calliper measurements of fluctuating asymmetry** ***ln (2|L−R|/(L+R))*** **between** ***Lycaon pictus*** **and** ***Canis mesomelas*** **.** Only traits exhibiting ideal FA area shown (see Tables S2 and S3).
(TIF)

Figure S3 **Log-transformed fluctuating asymmetry** ***ln (2|L−R|/(L+R))*** **in** ***Lycaon pictus*** **for (a) calliper measurements, and (b) photogrammetric measurements, plotted against time with independent linear regression fits for illustrative purposes.** Only those characters exhibiting ideal FA are shown (see Tables S3 and S4). Character labels for calliper measurements refer to those listed in Table S1. Character labels for photogrammetric measurements refer to those numbered 1 to 18 in Figure S1.
(ZIP)

Figure S4 **Diagnostic residual plots for regression of fluctuating asymmetry** ***ln(2|L−R|/(L+R))*** **against time for (a) calliper measurements, and (b) photogrammetric measurements.**
(ZIP)

Figure S5 **Diagnostic residual plots for regression of fluctuating asymmetry** ***ln(CFA)*** **against time for (a) calliper measurements, and (b) photogrammetric measurements.**
(ZIP)

Table S1 **Calliper measurements taken from** ***Lycaon pictus*** **skulls.** Measurements were taken to an accuracy of 0.01 mm, with the exception of the mandible measurement (*lmand*) which was measured to an accuracy of 0.5 mm using larger skull callipers.
(DOCX)

Table S2 **Tests for ideal FA in calliper measurements of** ***2 (L−R)/(L+R)*** **in** ***Canis mesomelas*** **skulls collected between 1949 and 2000.** Character names refer to those given in Table S1. Based on these tests characters are labelled as either 1: included, or 0: not included.
(DOCX)

Table S3 **Tests for ideal FA in calliper measurements of** ***2 (L−R)/(L+R)*** **in** ***Lycaon pictus*** **skulls collected between 1913 and 2001.** Character names refer to those given in Table S1. Based on these tests characters are labelled as either 1: included, or 0: not included.
(DOCX)

Table S4 **Tests for ideal FA in photogrammetric measurements of** ***2 (L−R)/(L+R)*** **in** ***Lycaon pictus*** **skulls collected between 1913 and 2001.** Character names refer to those numbered 1 to 18 in Figure S1. Based on these tests characters are labelled as either 1: included, or 0: not included.
(DOCX)

Table S5 **Analysis of variance table for fixed effects from mixed model linear regression fit to** ***ln (|FA|)*** **.** *p*-values were obtained using a parametric bootstrap, as described in the main text. The interaction term refers to that between *year* and *country* and was retained despite not being significant.
(DOCX)

Table S6 **Analysis of variance table for linear regression fit to (a)** ***ln (CFA)*** **, and (b) Haldanes.** The interaction between *year* (or *generation*) and *country* was retained despite not being significant.
(ZIP)
