# Supplementary figures and images for: Non-Adaptive Phenotypic Evolution of the Endangered Carnivore Lycaon pictus
Source: PLoS One. 2013 Sep 23;8(9):e73856. doi: 10.1371/journal.pone.0073856 (PMC3781135; doi:10.1371/journal.pone.0073856)

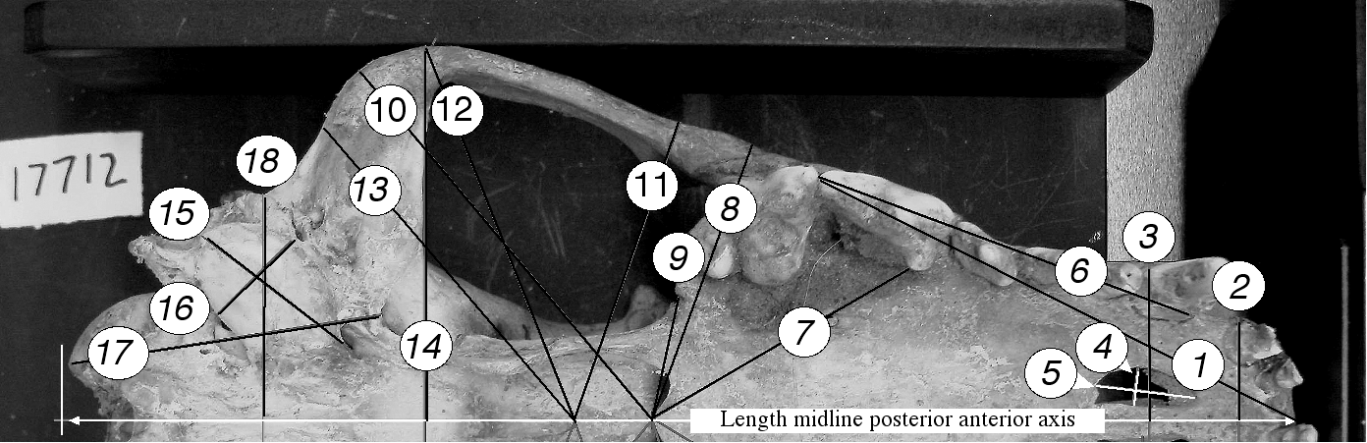

Supplement: Figure S1 — Photogrammetric measurements taken on Lycaon pictus skulls. Images were taken using a skull stand and spirit level, and eighteen paired photogrammetric measurements were taken from the underside of the cranium and analysed using Adobe Photoshop image software. (TIF) [file pone.0073856.s001.tif]

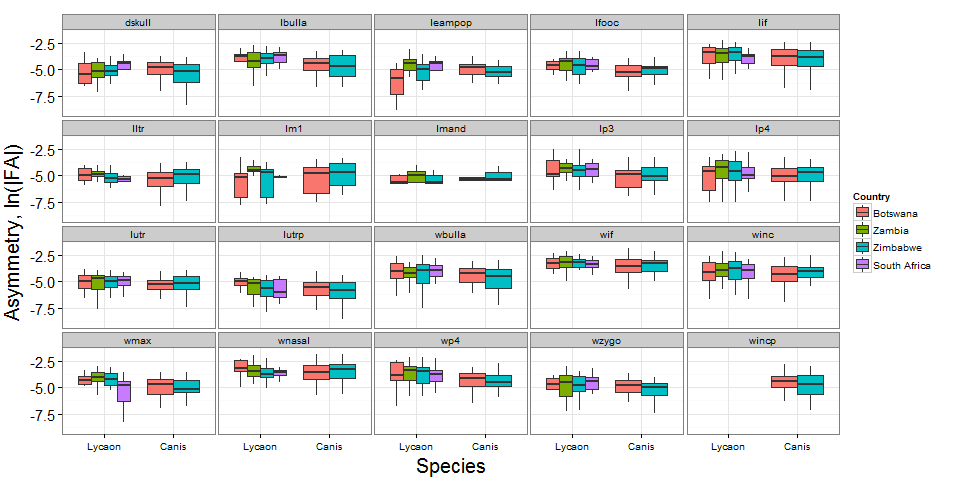

Supplement: Figure S2 — Comparison of log-transformed calliper measurements of fluctuating asymmetry ln (2|L−R|/(L+R)) between Lycaon pictus and Canis mesomelas . Only traits exhibiting ideal FA area shown (see Tables S2 and S3). (TIF) [file pone.0073856.s002.tif]

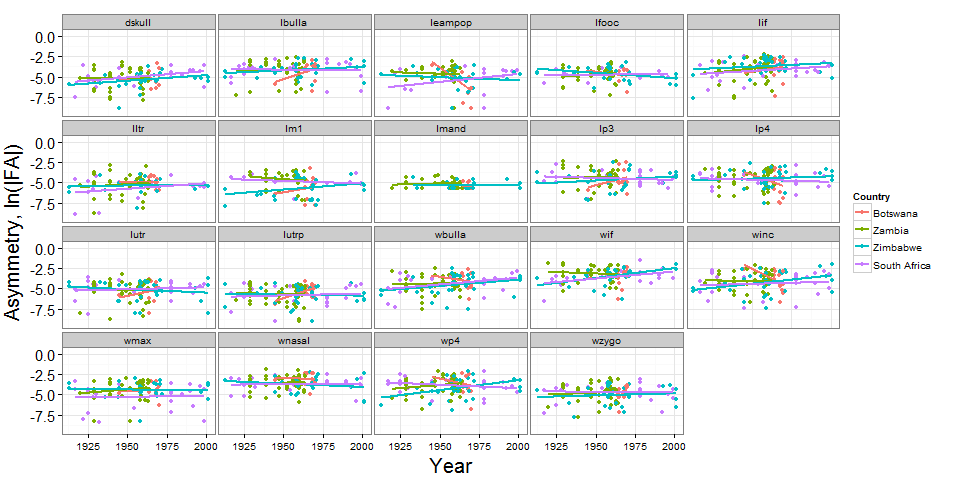

Supplement: Figure S3 — Log-transformed fluctuating asymmetry ln (2|L−R|/(L+R)) in Lycaon pictus for (a) calliper measurements, and (b) photogrammetric measurements, plotted against time with independent linear regression fits for illustrative purposes. Only those characters exhibiting ideal FA are shown (see Tables S3 and S4). Character labels for calliper measurements refer to those listed in Table S1. Character labels for photogrammetric measurements refer to those numbered 1 to 18 in Figure S1. (ZIP) [file pone.0073856.s003.zip › figure_S3a.tif]

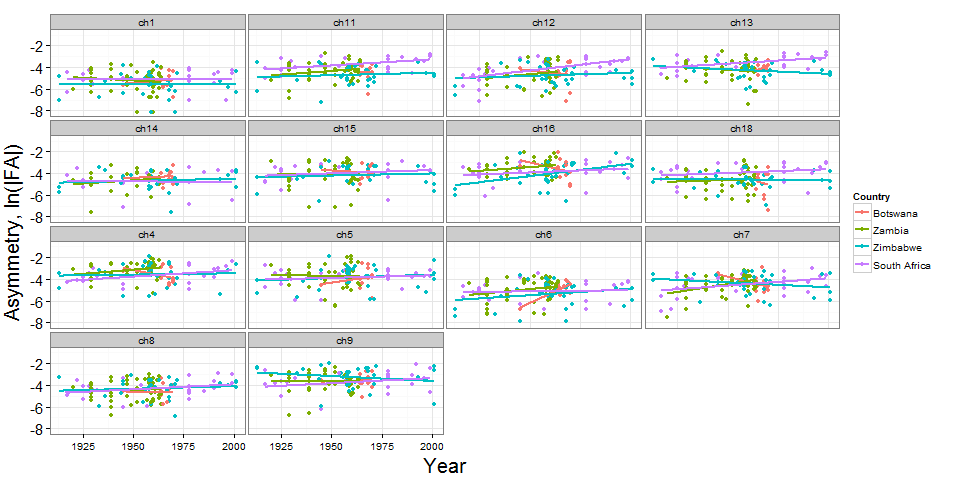

Supplement: Figure S3 — Log-transformed fluctuating asymmetry ln (2|L−R|/(L+R)) in Lycaon pictus for (a) calliper measurements, and (b) photogrammetric measurements, plotted against time with independent linear regression fits for illustrative purposes. Only those characters exhibiting ideal FA are shown (see Tables S3 and S4). Character labels for calliper measurements refer to those listed in Table S1. Character labels for photogrammetric measurements refer to those numbered 1 to 18 in Figure S1. (ZIP) [file pone.0073856.s003.zip › figure_S3b.tif]

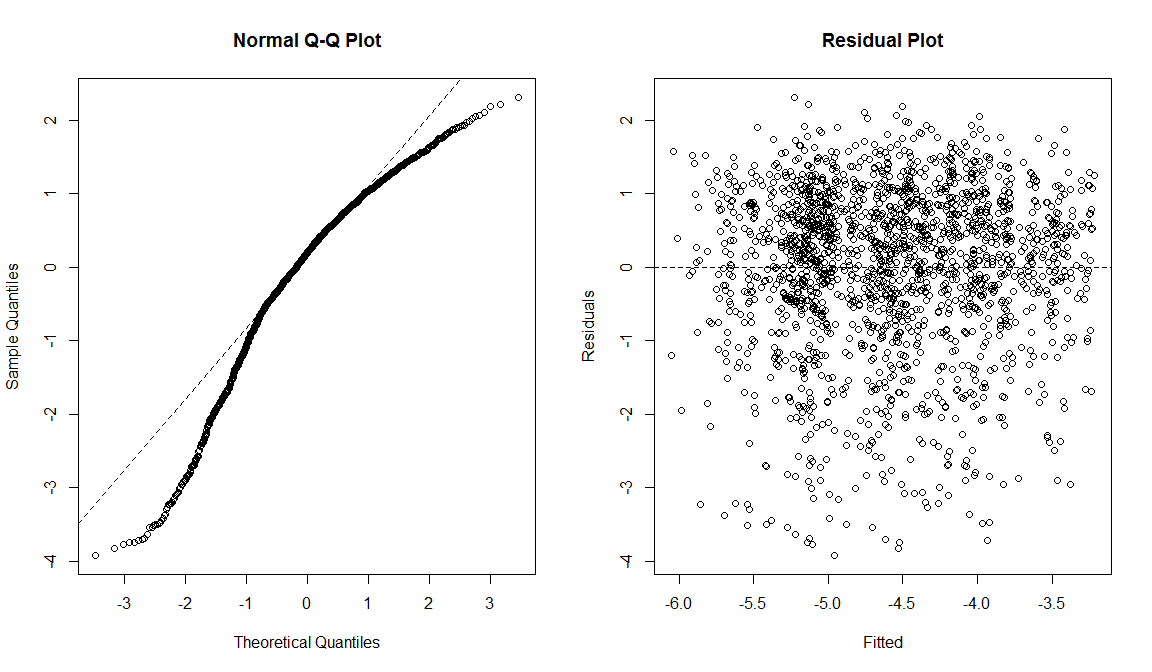

Supplement: Figure S4 — Diagnostic residual plots for regression of fluctuating asymmetry ln(2|L−R|/(L+R)) against time for (a) calliper measurements, and (b) photogrammetric measurements. (ZIP) [file pone.0073856.s004.zip › figure_S4a.tif]

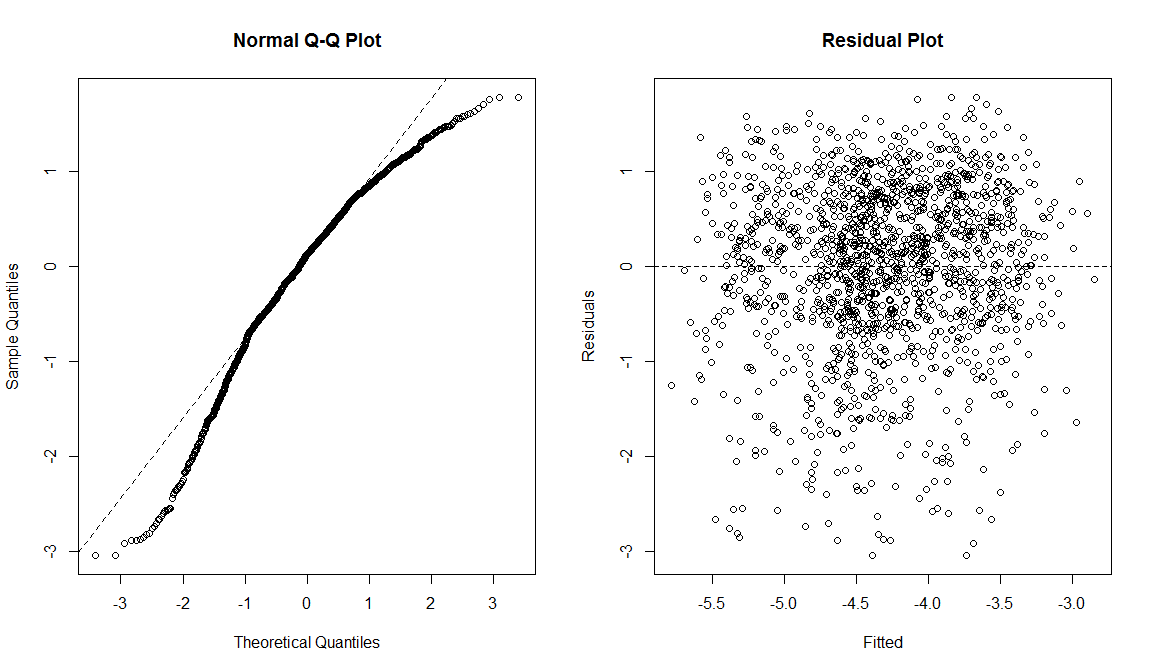

Supplement: Figure S4 — Diagnostic residual plots for regression of fluctuating asymmetry ln(2|L−R|/(L+R)) against time for (a) calliper measurements, and (b) photogrammetric measurements. (ZIP) [file pone.0073856.s004.zip › figure_S4b.tif]

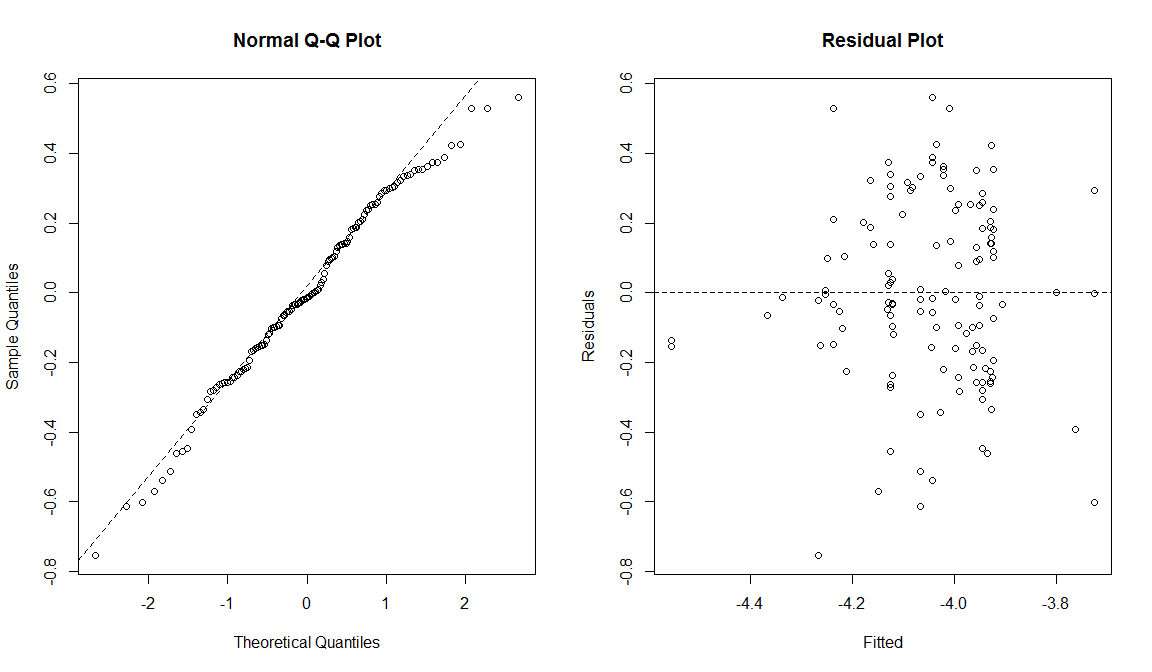

Supplement: Figure S5 — Diagnostic residual plots for regression of fluctuating asymmetry ln(CFA) against time for (a) calliper measurements, and (b) photogrammetric measurements. (ZIP) [file pone.0073856.s005.zip › figure_S5a.tif]

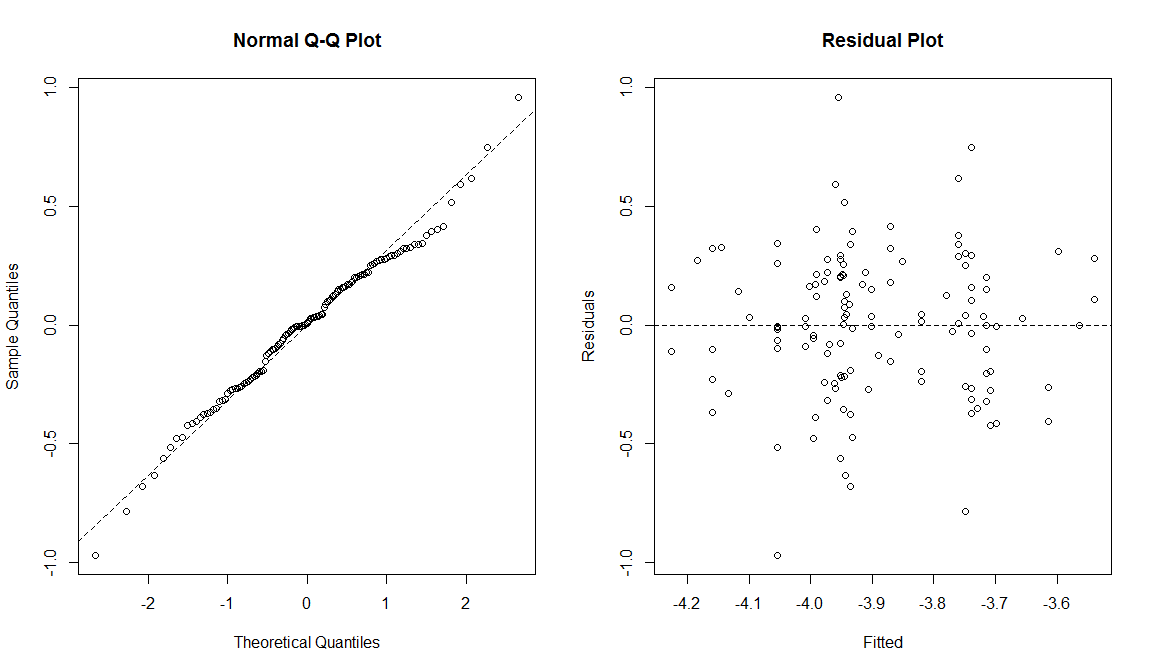

Supplement: Figure S5 — Diagnostic residual plots for regression of fluctuating asymmetry ln(CFA) against time for (a) calliper measurements, and (b) photogrammetric measurements. (ZIP) [file pone.0073856.s005.zip › figure_S5b.tif]
