# Supplementary material for: Non-Adaptive Phenotypic Evolution of the Endangered Carnivore Lycaon pictus
Source: PLoS One. 2013 Sep 23;8(9):e73856. doi: 10.1371/journal.pone.0073856 (PMC3781135; doi:10.1371/journal.pone.0073856)
Supplement: Table S1 — Calliper measurements taken from Lycaon pictus skulls. Measurements were taken to an accuracy of 0.01 mm, with the exception of the mandible measurement (lmand) which was measured to an accuracy of 0.5 mm using larger skull callipers. (DOCX) [file pone.0073856.s006.docx]

| Character | Description |
| --- | --- |
|  |  |
| winc | Width of the incisors: Median palate suture to outside of premaxillary element |
| wincp | Width of the incisors plus canines: Median palate suture to outside of canine socket on maxillary element |
| wnasals | Width of nasal bone elements: maximum measurement |
| wmax | Width of maxillary: Posterior point of the median palate suture to cleft dorsal to the first molar on the upper jaw |
| wzygo | Width of zygomatic arch: Tip of prosphenoid plate to maximum width of squamosal |
| lbulla | Length of auditory bulla: maximum length from medial lacerate foramen to posterior surface |
| wbulla | Width of auditory bulla: maximum measurement |
| dskull | Depth of skull: Squamosal ridge above external auditory meatus to suture joining parietal and frontal elements |
| lp3 | Maximum length of Upper P3 taken from base of tooth or bone socket |
| lp4 | Length of fourth upper premolar (upper carnassial) measured at base of tooth |
| wp4 | Width of fourth upper premolar: maximum measured at base of tooth |
| lutr | Length of upper tooth row: Posterior base of upper carnassial to anterior base of canine |
| lutrp | Length of upper tooth row plus incisors: Posterior base of upper carnassial to anterior base of first incisor |
| lmand | Length of mandible: Maximum length from condyle to anterior base of first incisor |
| lltr | Length of lower tooth row: Posterior surface of the first lower molar (lower carnassial) to anterior surface of the first lower premolar |
| lm1 | Length of lower carnassial taken from tooth socket on inside of jaw |
| lfooc | Length from posterior Foramen Ovale to Occipital Condyle |
| lif | Length of incisive foramen: maximum measurement |
| wif | Width of incisive foramen: maximum measurement |
| leampop | Length from external auditory meatus to post-orbital process from base of squamosal bone |
|  |  |
