# Supplementary material for: Non-Adaptive Phenotypic Evolution of the Endangered Carnivore Lycaon pictus
Source: PLoS One. 2013 Sep 23;8(9):e73856. doi: 10.1371/journal.pone.0073856 (PMC3781135; doi:10.1371/journal.pone.0073856)
Supplement: Table S2 — Tests for ideal FA in calliper measurements of 2 (L−R)/(L+R) in Canis mesomelas skulls collected between 1949 and 2000. Character names refer to those given in Table S1. Based on these tests characters are labelled as either 1: included, or 0: not included. (DOCX) [file pone.0073856.s007.docx]

| Character | Sample | Mean | *p*-value | adjusted | Skew | *p*-value | adjusted | Kurtosis | *p*-value | adjusted | Include |
| --- | --- | --- | --- | --- | --- | --- | --- | --- | --- | --- | --- |
|  | size |  |  | *p*-value |  |  | *p*-value |  |  | *p*-value |  |
|  |  |  |  |  |  |  |  |  |  |  |  |
| winc | 99 | 0.004 | 0.158 | 2.368 | 0.415 | 0.092 | 1.653 | 0.740 | 0.934 | 6.535 | 1 |
| wincp | 93 | 0.002 | 0.318 | 3.811 | -0.047 | 0.852 | 1.705 | 0.688 | 0.912 | 8.209 | 1 |
| wnasal | 101 | 0.002 | 0.702 | 4.916 | -0.626 | 0.010 | 0.204 | 1.180 | 0.992 | 3.969 | 1 |
| wmax | 99 | 0.001 | 0.399 | 4.389 | 0.024 | 0.922 | 0.922 | 0.624 | 0.897 | 8.975 | 1 |
| wzygo | 91 | 0.000 | 0.837 | 1.674 | -0.146 | 0.570 | 5.702 | -0.311 | 0.272 | 4.903 | 1 |
| lbulla | 88 | -0.003 | 0.105 | 1.788 | 0.121 | 0.642 | 3.852 | -0.243 | 0.321 | 5.451 | 1 |
| wbulla | 85 | -0.003 | 0.203 | 2.644 | -0.151 | 0.571 | 5.139 | -0.355 | 0.252 | 4.793 | 1 |
| dskull | 87 | 0.002 | 0.166 | 2.327 | -0.266 | 0.312 | 4.674 | -0.576 | 0.137 | 2.731 | 1 |
| lp3 | 101 | 0.000 | 0.990 | 0.990 | 0.202 | 0.406 | 4.471 | 2.908 | 1.000 | 2.000 | 1 |
| lp4 | 95 | -0.001 | 0.710 | 4.262 | -0.127 | 0.614 | 4.296 | 0.737 | 0.929 | 7.429 | 1 |
| wp4 | 97 | -0.001 | 0.761 | 3.044 | -0.209 | 0.401 | 4.813 | 0.096 | 0.577 | 7.498 | 1 |
| lutr | 95 | 0.002 | 0.054 | 1.076 | -0.090 | 0.719 | 2.876 | 0.293 | 0.720 | 8.641 | 1 |
| lutrp | 94 | 0.001 | 0.069 | 1.305 | -0.468 | 0.064 | 1.218 | 0.837 | 0.951 | 5.707 | 1 |
| lmand | 97 | 0.000 | 0.836 | 2.509 | -0.271 | 0.275 | 4.405 | 0.342 | 0.754 | 8.294 | 1 |
| lltr | 92 | 0.001 | 0.129 | 2.072 | 0.114 | 0.654 | 3.271 | -0.127 | 0.402 | 6.025 | 1 |
| lm1 | 93 | 0.000 | 0.718 | 3.592 | 0.304 | 0.231 | 3.933 | 10.306 | 1.000 | 1.000 | 1 |
| lfooc | 88 | 0.002 | 0.098 | 1.762 | -0.220 | 0.400 | 5.195 | -0.210 | 0.344 | 5.498 | 1 |
| lif | 99 | 0.003 | 0.438 | 3.942 | 0.213 | 0.386 | 5.405 | -0.106 | 0.415 | 5.807 | 1 |
| wif | 96 | 0.004 | 0.409 | 4.091 | 0.065 | 0.794 | 2.383 | 0.835 | 0.953 | 4.763 | 1 |
| leampop | 88 | 0.000 | 0.595 | 4.759 | -0.137 | 0.599 | 4.788 | 1.461 | 0.997 | 2.992 | 1 |
|  |  |  |  |  |  |  |  |  |  |  |  |
