# Supplementary material for: Non-Adaptive Phenotypic Evolution of the Endangered Carnivore Lycaon pictus
Source: PLoS One. 2013 Sep 23;8(9):e73856. doi: 10.1371/journal.pone.0073856 (PMC3781135; doi:10.1371/journal.pone.0073856)
Supplement: Table S3 — Tests for ideal FA in calliper measurements of 2 (L−R)/(L+R) in Lycaon pictus skulls collected between 1913 and 2001. Character names refer to those given in Table S1. Based on these tests characters are labelled as either 1: included, or 0: not included. (DOCX) [file pone.0073856.s008.docx]

|  |  |  |  |  |  |  |  |  |  |  |  |
| --- | --- | --- | --- | --- | --- | --- | --- | --- | --- | --- | --- |
| Character | Sample | Mean | *p*-value | adjusted | Skew | *p*-value | adjusted | Kurtosis | *p*-value | adjusted | Include |
|  | size |  |  | *p*-value |  |  | *p*-value |  |  | *p*-value |  |
|  |  |  |  |  |  |  |  |  |  |  |  |
| winc | 122 | -0.008 | 0.016 | 0.309 | 0.160 | 0.471 | 3.764 | 2.053 | 1.000 | 3.000 | 1 |
| wincp | 123 | 0.009 | 0.000 | 0.001 | -0.139 | 0.529 | 3.705 | -0.125 | 0.388 | 7.376 | 0 |
| wnasal | 120 | 0.009 | 0.027 | 0.494 | -0.067 | 0.765 | 3.060 | 1.010 | 0.988 | 8.892 | 1 |
| wmax | 124 | 0.000 | 0.852 | 2.557 | 0.079 | 0.719 | 3.597 | 0.370 | 0.800 | 11.195 | 1 |
| wzygo | 107 | -0.002 | 0.219 | 3.068 | 0.247 | 0.296 | 3.259 | 0.776 | 0.949 | 11.393 | 1 |
| lbulla | 103 | -0.006 | 0.043 | 0.730 | 0.328 | 0.174 | 2.792 | -0.107 | 0.413 | 7.014 | 1 |
| wbulla | 107 | 0.002 | 0.363 | 3.633 | -0.011 | 0.962 | 0.962 | 0.815 | 0.957 | 10.530 | 1 |
| dskull | 107 | 0.000 | 0.765 | 3.827 | -0.417 | 0.078 | 1.403 | 1.199 | 0.994 | 7.954 | 1 |
| lp3 | 125 | -0.001 | 0.627 | 3.761 | -0.308 | 0.160 | 2.715 | 3.909 | 1.000 | 2.000 | 1 |
| lp4 | 124 | -0.001 | 0.612 | 4.286 | -0.292 | 0.185 | 2.770 | 1.214 | 0.997 | 4.985 | 1 |
| wp4 | 123 | 0.005 | 0.180 | 2.697 | 0.218 | 0.324 | 3.242 | 0.827 | 0.969 | 9.694 | 1 |
| lutr | 124 | -0.001 | 0.516 | 4.131 | 0.602 | 0.006 | 0.124 | 0.718 | 0.949 | 12.333 | 1 |
| lutrp | 123 | -0.001 | 0.158 | 2.535 | -0.060 | 0.785 | 2.355 | -0.442 | 0.159 | 3.172 | 1 |
| lmand | 80 | 0.000 | 0.943 | 1.885 | 0.110 | 0.688 | 4.131 | 0.223 | 0.658 | 9.869 | 1 |
| lltr | 99 | -0.001 | 0.282 | 3.668 | 0.316 | 0.200 | 2.799 | 8.188 | 1.000 | 2.000 | 1 |
| lm1 | 103 | 0.000 | 0.778 | 3.113 | -0.037 | 0.878 | 1.756 | 1.274 | 0.996 | 5.975 | 1 |
| lfooc | 85 | 0.000 | 0.971 | 0.971 | -0.230 | 0.386 | 3.476 | -0.134 | 0.401 | 7.210 | 1 |
| lif | 122 | 0.004 | 0.296 | 3.558 | -0.241 | 0.277 | 3.319 | -0.064 | 0.443 | 7.083 | 1 |
| wif | 122 | -0.005 | 0.345 | 3.791 | -0.279 | 0.208 | 2.709 | 1.129 | 0.995 | 6.962 | 1 |
| leampop | 99 | -0.001 | 0.487 | 4.383 | -0.618 | 0.012 | 0.229 | 1.446 | 0.998 | 3.993 | 1 |
|  |  |  |  |  |  |  |  |  |  |  |  |
