# Supplementary material for: Non-Adaptive Phenotypic Evolution of the Endangered Carnivore Lycaon pictus
Source: PLoS One. 2013 Sep 23;8(9):e73856. doi: 10.1371/journal.pone.0073856 (PMC3781135; doi:10.1371/journal.pone.0073856)
Supplement: Table S4 — Tests for ideal FA in photogrammetric measurements of 2 (L−R)/(L+R) in Lycaon pictus skulls collected between 1913 and 2001. Character names refer to those numbered 1 to 18 in Figure S1. Based on these tests characters are labelled as either 1: included, or 0: not included. (DOCX) [file pone.0073856.s009.docx]

|  |  |  |  |  |  |  |  |  |  |  |  |
| --- | --- | --- | --- | --- | --- | --- | --- | --- | --- | --- | --- |
| Character | Sample | Mean | *p*-value | adjusted | Skew | *p*-value | adjusted | Kurtosis | *p*-value | adjusted | Include |
|  | size |  |  | *p*-value |  |  | *p*-value |  |  | *p*-value |  |
|  |  |  |  |  |  |  |  |  |  |  |  |
| ch1 | 124 | 0.000 | 0.557 | 2.784 | 0.401 | 0.068 | 1.025 | 0.501 | 0.873 | 5.235 | 1 |
| ch2 | 129 | -0.014 | 0.000 | 0.000 | 0.452 | 0.036 | 0.578 | 0.631 | 0.928 | 4.642 | 0 |
| ch3 | 128 | -0.007 | 0.003 | 0.055 | 0.035 | 0.873 | 0.873 | 0.696 | 0.946 | 2.838 | 0 |
| ch4 | 115 | 0.002 | 0.606 | 2.423 | -0.300 | 0.189 | 2.078 | 0.076 | 0.566 | 6.224 | 1 |
| ch5 | 119 | -0.001 | 0.773 | 1.547 | 0.177 | 0.431 | 1.725 | 2.035 | 1.000 | 1.000 | 1 |
| ch6 | 121 | 0.002 | 0.088 | 1.053 | -0.255 | 0.253 | 2.527 | -0.012 | 0.489 | 6.851 | 1 |
| ch7 | 122 | -0.002 | 0.383 | 2.299 | 0.470 | 0.034 | 0.582 | 0.018 | 0.516 | 6.193 | 1 |
| ch8 | 124 | 0.001 | 0.770 | 2.309 | 0.092 | 0.675 | 2.026 | -0.029 | 0.474 | 7.110 | 1 |
| ch9 | 118 | 0.004 | 0.341 | 2.728 | 0.234 | 0.299 | 2.694 | -0.136 | 0.382 | 6.491 | 1 |
| ch10 | 114 | 0.000 | 0.886 | 0.886 | 0.685 | 0.003 | 0.051 | 0.681 | 0.931 | 3.725 | 0 |
| ch11 | 108 | 0.004 | 0.051 | 0.659 | 0.208 | 0.376 | 2.259 | 0.395 | 0.799 | 6.393 | 1 |
| ch12 | 119 | 0.004 | 0.019 | 0.281 | 0.064 | 0.775 | 1.551 | -0.007 | 0.494 | 6.423 | 1 |
| ch13 | 111 | 0.003 | 0.175 | 1.745 | 0.204 | 0.381 | 1.905 | 0.444 | 0.830 | 5.813 | 1 |
| ch14 | 82 | -0.003 | 0.088 | 0.969 | 0.394 | 0.145 | 1.888 | -0.079 | 0.442 | 7.074 | 1 |
| ch15 | 101 | 0.003 | 0.351 | 2.457 | -0.221 | 0.364 | 2.550 | -0.330 | 0.249 | 4.480 | 1 |
| ch16 | 105 | -0.004 | 0.299 | 2.691 | -0.235 | 0.326 | 2.606 | 1.133 | 0.991 | 1.982 | 1 |
| ch17 | 117 | 0.011 | 0.006 | 0.091 | 0.346 | 0.126 | 1.768 | 0.097 | 0.585 | 5.847 | 0 |
| ch18 | 112 | 0.004 | 0.039 | 0.548 | 0.327 | 0.158 | 1.898 | 0.287 | 0.732 | 6.591 | 1 |
|  |  |  |  |  |  |  |  |  |  |  |  |
