# Supplementary material for: Non-Adaptive Phenotypic Evolution of the Endangered Carnivore Lycaon pictus
Source: PLoS One. 2013 Sep 23;8(9):e73856. doi: 10.1371/journal.pone.0073856 (PMC3781135; doi:10.1371/journal.pone.0073856)
Supplement: Table S5 — Analysis of variance table for fixed effects from mixed model linear regression fit to ln (|FA|) . p-values were obtained using a parametric bootstrap, as described in the main text. The interaction term refers to that between year and country and was retained despite not being significant. (DOCX) [file pone.0073856.s010.docx]

| Method | Variable | df | MSE | F value | *p*-value |
| --- | --- | --- | --- | --- | --- |
|  |  |  |  |  |  |
| Callipers | Year | 1 | 9.727 | 7.772 | 0.008 |
|  | Country | 3 | 6.781 | 5.418 | <0.0001 |
|  | Interaction | 3 | 0.319 | 0.255 | 0.873 |
|  | Residuals | 1915 | 1.252 |  |  |
|  |  |  |  |  |  |
| Photogrammetric | Year | 1 | 10.333 | 12.790 | 0.002 |
|  | Country | 3 | 3.489 | 4.318 | 0.011 |
|  | Interaction | 3 | 0.853 | 1.056 | 0.435 |
|  | Residuals | 1514 | 0.808 |  |  |
|  |  |  |  |  |  |
