# Supplementary material for: Non-Adaptive Phenotypic Evolution of the Endangered Carnivore Lycaon pictus
Source: PLoS One. 2013 Sep 23;8(9):e73856. doi: 10.1371/journal.pone.0073856 (PMC3781135; doi:10.1371/journal.pone.0073856)
Supplement: Table S6 — Analysis of variance table for linear regression fit to (a) ln (CFA) , and (b) Haldanes. The interaction between year (or generation) and country was retained despite not being significant. (ZIP) [file pone.0073856.s011.zip › table_S6a.docx]

| Method | Variable | df | MSE | F value | *p*-value |
| --- | --- | --- | --- | --- | --- |
|  |  |  |  |  |  |
| Callipers | Year | 1 | 1.320 | 17.881 | <0.0001 |
|  | Country | 3 | 0.260 | 3.528 | 0.017 |
|  | Interaction | 3 | 0.107 | 1.451 | 0.231 |
|  | Residuals | 122 | 0.074 |  |  |
|  |  |  |  |  |  |
| Photogrammetric | Year | 1 | 1.248 | 12.835 | <0.0001 |
|  | Country | 3 | 0.288 | 2.966 | 0.035 |
|  | Interaction | 3 | 0.238 | 2.445 | 0.067 |
|  | Residuals | 121 | 0.097 |  |  |
|  |  |  |  |  |  |
