# Supplementary material for: Non-Adaptive Phenotypic Evolution of the Endangered Carnivore Lycaon pictus
Source: PLoS One. 2013 Sep 23;8(9):e73856. doi: 10.1371/journal.pone.0073856 (PMC3781135; doi:10.1371/journal.pone.0073856)
Supplement: Table S6 — Analysis of variance table for linear regression fit to (a) ln (CFA) , and (b) Haldanes. The interaction between year (or generation) and country was retained despite not being significant. (ZIP) [file pone.0073856.s011.zip › table_S6b.docx]

| Method | Variable | df | MSE | F value | *p*-value |
| --- | --- | --- | --- | --- | --- |
|  |  |  |  |  |  |
| Callipers | Generation | 1 | 10.677 | 12.815 | <0.0001 |
|  | Country | 3 | 4.434 | 5.322 | 0.002 |
|  | Interaction | 3 | 1.122 | 1.347 | 0.262 |
|  | Residuals | 122 | 0.833 |  |  |
|  |  |  |  |  |  |
| Photogrammetric | Generation | 1 | 12.206 | 14.311 | <0.0001 |
|  | Country | 3 | 2.034 | 2.384 | 0.073 |
|  | Interaction | 3 | 2.162 | 2.535 | 0.060 |
|  | Residuals | 121 | 0.853 |  |  |
|  |  |  |  |  |  |
